# Occupational Factors Associated with Changes in the Body Mass Index of Korean Male Manual Workers

**DOI:** 10.1186/2052-4374-25-40

**Published:** 2013-12-27

**Authors:** In-Woong Song, Kuck-Hyun Woo, Jin-Seok Kim, Seong-Yong Yoon, Joo-Yong Na, Jin-Hyun Yu, Seong-Yong Cho

**Affiliations:** 1 Department of Occupational and Environmental Medicine, Soonchunhyang University Gumi Hospital, 179, Gongdan 1-dong, Gumi-si, Gyeongbuk 730-706, Republic of Korea

**Keywords:** Body mass index, Obesity, Occupations

## Abstract

**Objectives:**

This study was carried out to analyze and compare the occupational factors that could influence changes in body mass index (BMI) in male manual workers stratified into short-term and long-term work experience groups.

**Methods:**

The subjects were 299 male manual workers (sampled systematically) from 27 workplaces, who had undergone travelling medical examinations at a university hospital between March 28 and May 10, 2013, and had also undergone medical examinations at the same hospital in 2012. Their general and occupational characteristics were investigated through a structured, self-administered questionnaire. The BMI at each point in time was calculated based on the anthropometric results of the medical examinations. Multiple regression analyses were conducted on outcomes of the BMI change and predictors composed of the general and occupational characteristics, with the subjects stratified into groups with 5 years or less (short-term) versus more than 5 years (long-term) of work experience at the present post.

**Results:**

In the short-term work experience group, the BMI increases of 3-shift workers and groups reporting disagreement with feeling “insufficient job control” and “lack of reward” at work, two of the subscales of job stress, were significantly higher than those of daytime workers and high-stress groups, respectively. In the long-term work experience group, However, although the BMI increase for 3-shift workers was also significantly higher than that of daytime workers, none of the job stress factors were significantly associated with a BMI increase, whereas the social factors of education and marital status were significant, and some lifestyle factors (such as smoking and regular exercise) were also significant.

**Conclusion:**

This study showed that, except for 3-shift work, the factors associated with BMI increase could differ depending on the length of job experience. Consequently, different strategies may be needed for workers with short-term versus long-term job experience when designing interventions for preventing their obesity.

## Introduction

The obese have an elevated risk of all-cause mortality and of many specific diseases. Greatly increased relative risks (RR»3) among the obese for type 2 diabetes, gallbladder disease, dyslipidemia, metabolic syndrome, breathlessness, and sleep apnea; moderately increased relative risks (RR 2–3) for coronary heart disease, hypertension, osteoarthritis (in the knees and hips), hyperuricemia, and gout; and mildly increased relative risks (RR 1–2) for cancer, reproductive hormone abnormalities, polycystic ovary syndrome, impaired fertility, lower back pain, increased anesthetic risk, and fetal defects associated with maternal obesity have been reported by the WHO [1]. Obesity is also a risk factor for a fatty liver, cirrhosis of the liver, and chronic renal failure [2,3]. According to a meta-analysis revealing an association between body mass index (BMI) and cancer, an increase in BMI of 5 ㎏∕㎡ in males showed a strong relationship with esophageal adenocarcinoma, thyroid cancer, colon cancer, and renal cancer, and for females, endometrial cancer, gallbladder cancer, esophageal adenocarcinoma, and renal cancer [4]. Obesity might also increase risks of occupational diseases such as musculoskeletal disorders, cardiovascular disease, asthma, and vibration-induced injury. Moreover, obesity might modify physiological responses to neurotoxic substances and immunological responses to chemicals in the workplace, interact with job stress, and limit the wearing of personal protective devices and their effects [5].

The global change between 1980 and 2008 for men was 0.4 ㎏∕㎡ per decade, and for women, 0.5 ㎏∕㎡ per decade [6]. According to a study based on the medical examinations data of the National Health Insurance Corporation, Korean men’s and women’s BMI increased by an average of 1.0 ㎏∕㎡ and 0.7 ㎏∕㎡, respectively, from 1997 to 2007 [7]. The prevalence of obesity (for ages 19 and over, age-standardized) in Korea [8] for men increased from 25.1% in 1998 to 36.2% in 2007 and stayed at 36% or so for 3 years after that, and for women, stayed at 26% or so from 1998 to 2010. The rise in BMI and the prevalence of obesity will increase the social cost of the management and treatment of obesity and its related diseases [9]. Obesity will also increase workers’ absence from work and decrease labor productivity [10]. Not only that, but obesity also increases the amount of money that must be paid for workers’ compensation. Obese workers file more compensation claims, have more costly claims, and have more lost workdays than non-obese workers [11]. Although obesity is not caused just by occupation, its control is an important part of workers’ health management, due to the relationship it has with occupational and non-occupational diseases that can occur in workers.

Genetic factors and environmental factors are complexly intertwined in the occurrence of obesity [12]. Environmental factors include socioeconomic factors (such as education, occupation, and income) and lifestyle factors (such as physical activity, smoking, and drinking); these have been ascertained in many studies to be associated with obesity [13,14]. Occupational factors such as job stress, long working hours (including overtime work), shift work, and unemployment are also associated with obesity [5]. Shift work could have an impact on diet, exercise, and sleep, thereby inducing weight gain [15]. Job stress might affect lifestyle, such as dietary habits and physical activity, thereby contributing to weight gain [16-18]; however, it might also decrease appetite and so contribute to weight loss [19,20].

It is very important to fully grasp the occupational risk factors of obesity in terms of the efficient prevention and systematic management of workers’ obesity. To date, there have been many reports on the association of occupational factors with obesity, and so the risk factors have been revealed, but there have been few reports regarding the influence of the length of workers’ job experience on the related factors. This study was carried out to analyze the occupational factors that could influence BMI change in male manual workers (stratified into a group with only short-term experience and one with long-term experience working at the present post) and to ascertain whether there is any difference among the associated factors between the groups.

## Materials and methods

### Subjects

The sample was selected from among the manual workers of 27 workplaces who had undergone travelling medical examinations conducted by a university hospital between March 28 and May 10, 2013, and had also gotten medical examinations conducted by the same hospital in 2012. First, 387 workers were sampled via a systematic sampling method in which one person was selected randomly among the first 10 in order of receipt number, and each 10th subsequent patient was also selected. Among the 387 workers, 6 who were suffering from diabetes mellitus were ruled out because it could give rise to weight loss. The data on the remaining 381 people were analyzed, but there was no statistical significance in BMI change for the female subjects, and so, ultimately, only the data on the male subjects were analyzed. Accordingly, in the end, the subjects studied were 299 male manual workers.

### Methods

General and occupational characteristics were investigated through a structured, self-administered questionnaire. The general characteristics included age, education, marital status, coffee drinking, and amount of sleep. Workers were asked to select one of four choices from “elementary school”, “middle school”, “high school”, and “college or higher” to indicate their level of education completed. They were also asked to select one of four choices (“single”, “married”, “divorced”, and “widowed”) to indicate their marital status. Workers reported the number of cups of coffee they consumed per day and the number of hours of sleep they got as well. The lifestyle factors of smoking, drinking, and physical activity were investigated through separate self-administered questionnaires which had been included in the 2012 and 2013 medical examinations. In the questionnaires, workers had to select one of three choices (“not smoking”, “quit smoking”, and “currently smoking”) to indicate their present smoking state. Workers reported the amount of their drinking as the number of glasses of alcohol consumed per week, irrespective of the type of alcohol. They were also asked to indicate the type and frequency of physical activity they engaged in. Those who engaged in strenuous physical activity for at least 20 minutes a day for at least 3 days for the past week were defined as performing high-intensity exercise, whereas those who engaged in less strenuous, moderate physical activity for at least 30 minutes a day for at least 5 days for the past week were defined as performing moderate-intensity exercise. Performers of both high- and moderate-intensity exercise were regarded as getting regular exercise.

The occupational characteristics we investigated included working hours, shift work, sitting time (a percentage of the workday), job stress, and experience at the present post. Workers were asked to report the number of hours they spent working and select one of three choices (“no shift work”, “2 shifts”, and “3 shifts”) to indicate their shift work. They had to select one of four choices (“0-24%”, “25-49%”, “50-74%”, and “75-100%”) to indicate the percentage of their workday spent sitting. Job stress was measured with the short form of the Korean Occupational Stress Scale (KOSS-SF) [21]. This tool for measuring job stress consists of 7 factors: job demand, insufficient job control, inadequate social support, job insecurity, organizational injustice, lack of reward, and poor occupational climate. Each factor with regard to job stress is scored on the basis of 100 points, and the higher the score, the more stressed workers were with their job. The subjects were divided into lower 50% and higher 50% groups to indicate the low and high job stress groups, based on the median of the KOSS-SF reference value (for men) [21]. They reported the length of time they had worked at their current position in the workplace in years and months to indicate the length of their experience at the present post. The BMIs for 2012 and 2013 were calculated, using the formula (body weight)/(height)^2^ based on the anthropometric results of the annual medical examinations.

### Analysis

Some of the investigated variables were converted into appropriate categories to be applied to the analyses. Specifically, education was divided into two categories: “high school or lower” and “college or higher.” Smoking was divided into four categories (“never or formerly smoked”, “started or resumed smoking”, “quit smoking”, and “continued smoking”) based on smoking status in 2012 and 2013. “Never or formerly smoked” corresponds to the case of not having smoked during the observation period, that is, as reported at both examinations. “Started or resumed smoking” corresponds to the case in which “not smoking” or “quit smoking” was reported in 2012 but “currently smoking” was reported in 2013. “Quit smoking” corresponds to the case in which “currently smoking” in 2012 became “not smoking” or “quit smoking” in 2013. “Continued smoking” corresponds to those having smoked throughout the observation period. The weekly amounts of drinking in 2012 and 2013 were compared, and drinking was divided into four categories: “non-drinking”, “no change in drinking”, “decrease in drinking”, and “increase in drinking”. Regular exercise was divided into four categories (“not performed”, “stopped performing”, “began performing”, and “continued performing”), based on the states of physical activity reported in 2012 and 2013. The amount of sleep and working hours were divided into two categories (“less than 7 hours” and “7 hours or more”) and three categories (“9 hours or less”, “more than 9 hours but less than 12 hours”, and “12 hours or more”), respectively.

All of the analyses were conducted with the workers’ data stratified into those with “5 years or less” experience and “more than 5 years” experience at the current job, indicating the short- and long-term work groups, respectively, on the basis of a threshold of 5 years, which was the median value of the workers’ experience at the present post. First, the means and standard deviations for the continuous variables of age and coffee drinking were calculated. Second, frequency analyses for the categorical variables of education, marital status, smoking, drinking, regular exercise, amount of sleep, working hours, shift work, sitting time (a percentage of the workday), and job stress were conducted; the means and standard deviations of the BMI change for each value of the variables were presented. Third, the means and standard deviations of the BMIs in 2012 and 2013 and the change in BMI between them were presented. Paired samples t-tests were conducted to compare the corresponding two years’ BMI means for the short- and long-term work groups and the whole sample, respectively. Finally, multiple regression analyses were conducted on outcomes of the BMI change and predictors composed of the general and occupational characteristics. The SPSS 14.0 program for Windows (IBM Corporation, Armonk, NY, USA) was used for the statistical analyses. The level of statistical significance applied to the analyses was less than 0.05.

## Results

### General and occupational characteristics of the subjects

The general characteristics of the subjects for the groups of “5 years or less” and “more than 5 years” working at the present post are shown in Table 1. The mean ages of the short- and long-term work groups were 32.6 and 38.3, and so the mean age of the long-term work group was 5.7 more than that of the short-term work group. With respect to the level of education, the frequency of “college or higher” (63.6%) was higher than that of “high school or lower” (36.4%) for the short-term work group, whereas the frequency of “high school or lower” (62.2%) was higher than that of “college or higher” (37.8%) for the long-term work group. The frequency of married men for the short- and long-term work groups (53.6% and 76.4%) was higher than that of single or divorced men; there were no widowed men among the subjects. The frequency of “continued smoking” (43.7%) was the highest, and that of “never or formerly smoked” (42.4%) the second highest in the short-term work group. Similarly, the frequency of “continued smoking” (46.6%) was the highest, and that of “never or formerly smoked” (41.2%) the second highest in the long-term work group. The frequency of “decrease in drinking” (39.7%) was the highest, and that of “increase in drinking” (30.5%) the second highest in the short-term work group. Likewise, the frequency of “decrease in drinking” (38.5%) was the highest, and that of “increase in drinking” (27.7%) the second highest in the long-term work group. With respect to regular exercise, the frequencies of “not performed” were the highest in the short- and long-term work groups alike (76.8% and 69.6%, respectively). The average amounts of coffee drinking in the short- and long-term work groups were 2.4 and 2.7 cups a day, respectively. The frequencies of “less than 7 hours” of sleep in the short- and long-term work groups (53.0% and 53.4%, respectively) were a bit higher than those of “7 hours or more” (47.0% and 46.6%, respectively).

**Table 1:** General characteristics of the subjects

**Variables**	5 years or less*			More than 5 years*		
	**Mean ± SD (kg/m**^**2**^**)**	**No(%)**	**BMI change (Mean ± SD, kg/m**^**2**^**)**	**Mean ± SD (kg/m**^**2**^**)**	**No(%)**	**BMI change (Mean ± SD, kg/m**^**2**^**)**
Age (years)	32.60 ± 6.52			38.31 ± 7.23		
Education						
High school or lower		55(36.4)	0.43 ± 1.30		92(62.2)	0.40 ± 1.06
College or higher		96(63.6)	0.38 ± 0.90		56(37.8)	0.06 ± 0.93
Marital status						
Single or divorced		70(46.4)	0.41 ± 1.09		35(23.6)	0.08 ± 1.05
Married		81(53.6)	0.39 ± 1.04		113(76.4)	0.33 ± 1.01
Smoking^†^						
Never or formerly smoked		64(42.4)	0.31 ± 1.00		61(41.2)	0.28 ± 1.00
Started or resumed smoking		8( 5.3)	0.57 ± 1.58		2( 1.4)	−0.39 ± 0.49
Quit smoking		7( 4.6)	0.43 ± 1.15		7( 4.7)	1.12 ± 1.01
Continued smoking		66(43.7)	0.52 ± 1.07		69(46.6)	0.20 ± 1.06
Alcohol drinking^‡^						
Non-drinking		17(11.3)	0.13 ± 0.82		20(13.5)	0.42 ± 1.17
No change		22(14.6)	0.50 ± 0.80		21(14.2)	0.39 ± 0.70
Decrease in drinking		60(39.7)	0.45 ± 1.05		57(38.5)	0.30 ± 1.16
Increase in drinking		46(30.5)	0.46 ± 1.27		41(27.7)	0.11 ± 0.96
Regular exercise^§^						
Not performed		116(76.8)	0.39 ± 0.98		103(69.6)	0.18 ± 0.94
Stopped performing		12( 7.9)	0.48 ± 1.31		15(10.1)	0.55 ± 0.88
Began performing		17(11.3)	0.56 ± 1.39		23(15.5)	0.56 ± 1.42
Continued performing		6( 4.0)	0.11 ± 1.17		7( 4.7)	−0.02 ± 0.71
Coffee drinking (cups/day)	2.41 ± 1.54			2.74 ± 1.69		
Amount of sleep						
< 7 hours		80(53.0)	0.42 ± 1.03		79(53.4)	0.24 ± 0.94
≥ 7 hours		71(47.0)	0.38 ± 1.09		69(46.6)	0.31 ± 1.11

* Length of experience working at the present post.

† The change in smoking status between 2012 and 2013.

‡ The change in the weekly amount of alcohol drinking between 2012 and 2013.

§ The change in regular exercise performance between 2012 and 2013. Those performing high- or moderate-intensity exercise were regarded as getting regular exercise.

Occupational characteristics of the subjects for the groups of “5 years or less” and “more than 5 years” working at the present post are presented in Table 2. The frequencies of “more than 9 hours but less than 12 hours” spent working were the highest in the short- and long-term work groups (49.0% and 52.7%, respectively). With respect to shift work, the frequencies of “no shift work” for the short- and long-term work experience groups were the highest (75.5% and 60.8%, respectively), and those of “3 shifts” the second highest (17.2% and 26.4%, respectively). The frequencies of “25-49%” of the time spent sitting during their workday were the highest for both the short- and long-term work groups (33.1% and 36.5%, respectively). The frequencies of the low job stress group with regard to all of the 7 factors were higher than those of the high stress group for the short- and long-term work experience groups alike.

**Table 2:** Occupational characteristics of the subjects

**Variables**		5 years or less*		More than 5 years*	
		**No(%)**	**BMI change**^**†**^	**No(%)**	**BMI change**^**†**^
Working hours	≤ 9 hours	44(29.1)	0.29 ± 1.04	36(24.3)	0.31 ± 0.85
	> 9 hours, < 12 hours	74(49.0)	0.33 ± 1.07	78(52.7)	0.21 ± 1.09
	≥ 12 hours	33(21.9)	0.71 ± 1.02	34(23.0)	0.37 ± 1.05
Shift work	No shift work	114(75.5)	0.35 ± 0.98	90(60.8)	0.23 ± 1.07
	Two shifts	11( 7.3)	0.50 ± 1.24	19(12.8)	0.29 ± 0.84
	Three shifts	26(17.2)	0.58 ± 1.30	39(26.4)	0.36 ± 1.02
Sitting time (% of workday)	0-24%	26(17.2)	0.43 ± 1.20	33(22.3)	0.37 ± 0.93
	25-49%	50(33.1)	0.38 ± 0.92	54(36.5)	0.04 ± 1.08
	50-74%	47(31.1)	0.25 ± 0.89	37(25.0)	0.40 ± 0.92
	75-100%	27(17.9)	0.74 ± 1.35	24(16.2)	0.45 ± 1.14
Job stress					
Job demand	Low	84(55.6)	0.45 ± 1.16	101(68.2)	0.22 ± 1.01
	High	63(41.7)	0.36 ± 0.93	46(31.1)	0.42 ± 1.04
Insufficient job control	Low	102(67.5)	0.56 ± 1.06	102(68.9)	0.25 ± 0.96
	High	45(29.8)	0.10 ± 1.03	45(30.4)	0.34 ± 1.16
Inadequate social support	Low	100(66.2)	0.46 ± 1.10	107(72.3)	0.18 ± 1.03
	High	46(30.5)	0.31 ± 1.00	40(27.0)	0.55 ± 0.96
Job insecurity	Low	110(72.8)	0.39 ± 1.10	113(76.4)	0.23 ± 0.96
	High	36(23.8)	0.49 ± 0.97	33(22.3)	0.44 ± 1.21
Organizational injustice	Low	105(69.5)	0.43 ± 1.10	109(73.6)	0.17 ± 0.98
	High	41(27.2)	0.36 ± 1.00	37(25.0)	0.60 ± 1.07
Lack of reward	Low	102(67.5)	0.57 ± 1.13	97(65.5)	0.15 ± 0.98
	High	45(29.8)	0.07 ± 0.82	50(33.8)	0.53 ± 1.06
Poor occupational climate	Low	76(50.3)	0.45 ± 1.21	85(57.4)	0.24 ± 0.95
	High	70(46.4)	0.37 ± 0.89	61(41.2)	0.33 ± 1.12

* Length of experience working at the present post.

† Mean ± SD, ㎏∕㎡.

### BMI change and its association with occupational factors

The average BMI in 2013 increased by 0.34 ㎏∕㎡ from 2012 for the whole sample. For the short- and long-term work experience groups, the average BMIs in 2013 increased by 0.40 ㎏∕㎡ and 0.27 ㎏∕㎡ respectively, compared with those in 2012 (Table 3).

**Table 3:** Body mass indices in 2012 and 2013 and their change by length of work experience

**Subjects**	**No(%)**	**BMI (Mean ± SD, kg/m**^**2**^**)**		**BMI change (Mean ± SD, kg/m**^**2**^**)**
		**2012**	**2013**	
5 years or less*	151(50.5)	24.59 ± 3.24	25.00 ± 3.39	0.40 ± 1.06^†^
more than 5 years*	148(49.5)	24.23 ± 2.88	24.50 ± 2.95	0.27 ± 1.02^†^
Total	299(100.0)	24.41 ± 3.06	24.75 ± 3.19	0.34 ± 1.04^†^

* Length of experience working at the present post.

† p-value: < 0.05.

As a result of the multiple regression analyses conducted on outcomes of the BMI increase and predictors composed of the general and occupational characteristics, the variables of 3-shift work, insufficient job control, and lack of reward were significant for the short-term work group (Table 4). The BMI increase for 3-shift workers was significantly higher than the corresponding increase for daytime workers. The BMI increases for low job stress groups with regard to the factors of “insufficient job control” and “lack of reward” were significantly higher than those for high stress groups. In other words, the BMI increases for 3-shift workers and those who were had sufficient job control and were properly rewarded were relatively high.

**Table 4:** Factors associated with change in BMI for the short-term work group*

	**Variables**	**B**	**SE**^**†**^	**β**	**t**	**p-value**
Age		−0.02	0.02	−0.12	−1.13	0.263
Education	High school or lower (Ref)					
	College or higher	−0.06	0.22	−0.03	−0.28	0.783
Marital status	Single or divorced (Ref)					
	Married	0.06	0.22	0.03	0.29	0.771
Smoking^‡^	Never or formerly smoked (Ref)					
	Started or resumed smoking	0.50	0.48	0.10	1.05	0.298
	Quit smoking	0.27	0.46	0.06	0.59	0.558
	Continued smoking	0.25	0.23	0.12	1.09	0.279
Alcohol drinking^§^	Non-drinking (Ref)					
	No change	0.29	0.40	0.10	0.72	0.470
	Decrease in drinking	0.21	0.34	0.10	0.62	0.533
	Increase in drinking	0.16	0.37	0.07	0.43	0.670
Regular exercise^∥^	Not performed (Ref)					
	Stopped performing	0.11	0.36	0.03	0.32	0.751
	Began performing	0.16	0.31	0.05	0.51	0.608
	Continued performing	0.35	0.55	0.06	0.64	0.525
Coffee drinking		0.01	0.07	0.02	0.16	0.873
Amount of sleep	< 7 hours (Ref)					
	≥ 7 hours	−0.04	0.20	−0.02	−0.19	0.851
Working hours	≤ 9 hours (Ref)					
	> 9 hours, < 12 hours	0.34	0.25	0.16	1.35	0.181
	≥ 12 hours	0.61	0.32	0.24	1.88	0.062
Shift work	No shift work (Ref)					
	Two shifts	0.50	0.42	0.13	1.18	0.239
	Three shifts	0.73	0.34	0.26	2.13	0.035
Sitting time	0-24% (Ref)					
	25-49%	0.07	0.33	0.03	0.20	0.842
	50-74%	−0.06	0.37	−0.02	−0.15	0.883
	75-100%	0.64	0.39	0.23	1.65	0.101
Job stress	Job demand	−0.20	0.23	−0.09	−0.86	0.393
	Insufficient job control	−0.55	0.24	−0.24	−2.29	0.024
	Inadequate social support	0.01	0.24	0.01	0.06	0.956
	Job insecurity	0.33	0.25	0.13	1.33	0.186
	Organizational injustice	0.10	0.26	0.04	0.40	0.690
	Lack of reward	−0.52	0.26	−0.22	−2.01	0.047
	Poor occupational climate	0.07	0.23	0.03	0.30	0.768

Dependent variable: The change in body mass indices between 2012 and 2013.

R^2^ = 0.229.

* Experience of 5 years or less working at the present post.

† Standard error.

‡ The change in smoking status between 2012 and 2013.

§ The change in the weekly amount of alcohol drinking between 2012 and 2013.

∥ The change in regular exercise performance between 2012 and 2013. Those performing high- or moderate-intensity exercise were regarded as getting regular exercise.

For the long-term work group, the variables of education, marital status, some factors involving smoking and regular exercise, and 3-shift work were significant (Table 5). The BMI increase for those with a level of education of high school or lower was significantly higher than that for those with a level of education of college or higher. The BMI increase for married men was significantly higher than that for single or divorced men. The BMI increase for those who had started or resumed smoking over the past year was significantly lower than that for those who had never smoked or had formerly smoked. The BMI increase for those who had stopped performing regular exercise for the past year was significantly higher than that for those who had not performed regular exercise. The BMI increase for 3-shift workers was significantly higher than that for daytime workers, as in the short-term work group. In other words, the BMI increases for those less educated, married men, those who had stopped performing regular exercise, and 3-shift workers were relatively high, and that for those who had started or resumed smoking was relatively low.

**Table 5:** Factors associated with BMI change for the long-term work group*

	**Variables**	**B**	**SE**^**†**^	**β**	**t**	**p-value**
Age		−0.01	0.02	−0.10	−0.84	0.403
Education	High school or lower (Ref)					
	College or higher	−0.66	0.22	−0.31	−3.03	0.003
Marital status	Single or divorced (Ref)					
	Married	0.49	0.23	0.20	2.13	0.035
Smoking^‡^	Never or formerly smoked (Ref)					
	Started or resumed smoking	−1.57	0.78	−0.18	−2.02	0.046
	Quit smoking	0.81	0.41	0.17	1.96	0.053
	Continued smoking	−0.20	0.19	−0.10	−1.02	0.309
Alcohol drinking^§^	Non-drinking (Ref)					
	No change	0.05	0.34	0.02	0.15	0.884
	Decrease in drinking	0.07	0.30	0.03	0.24	0.810
	Increase in drinking	−0.51	0.29	−0.22	−1.73	0.086
Regular exercise^∥^	Not performed (Ref)					
	Stopped performing	0.67	0.32	0.20	2.10	0.038
	Began performing	0.24	0.24	0.09	1.00	0.321
	Continued performing	−0.06	0.40	−0.01	−0.15	0.880
Coffee drinking		0.09	0.06	0.15	1.59	0.115
Amount of sleep	< 7 hours (Ref)					
	≥ 7 hours	0.03	0.18	0.01	0.17	0.865
Working hours	≤ 9 hours (Ref)					
	> 9 hours, < 12 hours	0.07	0.24	0.04	0.31	0.761
	≥ 12 hours	0.20	0.31	0.08	0.64	0.524
Shift work	No shift work (Ref)					
	Two shifts	−0.13	0.33	−0.04	−0.40	0.693
	Three shifts	0.58	0.27	0.24	2.16	0.033
Sitting time	0-24% (Ref)					
	25-49%	−0.29	0.24	−0.13	−1.18	0.242
	50-74%	0.10	0.28	0.04	0.35	0.729
	75-100%	0.53	0.35	0.19	1.54	0.126
Job stress	Job demand	0.01	0.20	0.00	0.03	0.978
	Insufficient job control	−0.08	0.22	−0.04	−0.38	0.705
	Inadequate social support	0.44	0.23	0.19	1.93	0.056
	Job insecurity	0.28	0.22	0.11	1.28	0.204
	Organizational injustice	0.05	0.24	0.02	0.21	0.831
	Lack of reward	0.30	0.21	0.14	1.40	0.163
	Poor occupational climate	0.10	0.19	0.05	0.54	0.587

Dependent variable: The change in body mass indices between 2012 and 2013.

R^2^ = 0.329.

* Experience of more than 5 years working at the present post.

† Standard error.

‡ The change in smoking status between 2012 and 2013.

§ The change in the weekly amount of alcohol drinking between 2012 and 2013.

∥ The change in regular exercise performance between 2012 and 2013. Those performing high- or moderate-intensity exercise were regarded as getting regular exercise.

## Discussion

The analyses of the factors associated with BMI increases showed that 3-shift work, insufficient job control, and lack of reward influenced the BMI increase in the short-term work group, while 3-shift work, education, marital status, and some factors involving smoking and regular exercise did so for the long-term work group. As for occupational factors, 3-shift work was positively associated with a BMI increase for both groups, and insufficient job control and lack of reward were negatively associated with a BMI increase just for the short-term work group. 3-shift workers had a higher BMI increase than daytime workers, and workers who were under sufficient job control and were properly rewarded had a higher BMI increase than workers who were not. On the other hand, social factors, such as education and marital status, and some lifestyle factors involving smoking and regular exercise, were associated with a BMI increase just for the long-term work group.

Recent studies have consistently showed a positive association between shift work and BMI or a BMI increase for male manual workers, like the results of this study [22-24]. In an observational study of a cohort of male workers working at a Japanese steel company, the odds ratios of shift work versus daytime work for 5%, 7.5%, and 10% BMI increases were 1.14, 1.13, and 1.13, and so the effect size of shift work for the BMI increase was presumed not to be large [25]. The change in eating habits (such as high-calorie intake, less frequent meals, and late last meals), the decrease in physical activity, and the change in sleeping habits (such as frequent and long naps) are thought to be the probable mechanisms for the weight gain resulting from shift work [15]. The change in hormone secretions is also assumed to contribute to weight gain [26,27]. Yamada et al. hypothesized that shift work could result in fatigue and inhibit the behaviors that prevent weight gain and the accumulation of abdominal fat [28]. In this study, 3-shift work was associated with a BMI increase, but 2-shift work was not. However, the number of 2-shift workers for the short- and long-term work groups were 11 and 19 people, respectively, and so they were too few to have sufficient test power. Thus, whether there is an association between 2-shift work and a BMI increase is unclear from the results of this study; additional research targeting 2-shift workers is needed.

The existing studies dealing with the association of job stress with weight or BMI have reported inconsistent results on positive associations, negative associations, and factors with no association [29-34]. A meta-analysis in Europe reported that both weight gain and weight loss were found to be associated with the onset of job stress, consistent with a U-shaped cross-sectional association between job stress and BMI. However, these associations were relatively modest [35]. One of the reasons for looking at various results of BMI changes against job stress is that the influence of stress on body weight could be bi-directional. Stress has an effect on eating habits and weight change (which is also influenced by sex, basal BMI, and the reactivity to cortisol and the like); the direction of weight change is decided based on these factors [19,20,36]. For example, when stressed, a man whose body weight is originally low suffers from a decrease in appetite and weight loss, whereas a man whose body weight is originally high suffers from an increase in appetite and weight gain. Consequently, stress could result in weight loss, weight gain, or no change, depending on the subject. In addition, the response of weight change to stress could be different according to the measurement tools of job stress used, and so it is necessary to use tools that have been verified as valid and reliable.

Because job control and reward could lead to psychological loosening in terms of time and money, there is a possibility that this psychological loosening will enable those who are under sufficient job control and are properly rewarded to gain weight. If people are under heavy stress, they put active coping mechanisms to work, which raise the ability to cope with difficult jobs. Then as time goes by, the negative effects of stress improve, and the coping mechanisms weaken [37]. Likewise, because job stress is a psychosocial problem associated with the adaptation to a work environment, it is presumed that the short-term work group would be more sensitive to it, and its effect would gradually disappear as experience increases. Thus, it is likely that there was a negative association of a BMI increase with the factors of insufficient job control and lack of reward with regard to job stress just for the short-term work group in this study.

The limitations of this study include using time spent sitting on duty to reflect work intensity instead of directly measuring it or investigating the workers’ jobs; not considering economic status or eating habits that are generally known to have an influence on BMI; not ruling out the effect of the factors at workplace level, such as the size of companies, the welfare system, and the presence of obesity prevention programs; and the relatively short observation period. However, to consider in the same analysis the occupational factors (such as shift work, job stress, and working hours), which are thought to be associated with a BMI change, and to compare the difference in the associated factors between the short- and long-term work groups are the merits of this study.

This study shows that, except for 3-shift work, the factors associated with BMI increase for the short- and long-term work groups could differ from each other. Consequently, it is necessary to use other strategies based on short- and long-term work groups when preparing measures to prevent workers’ obesity.

## Competing interests

The authors declare that they have no competing interests.

## Authors’ contributions

All authors had access to the data and played a role in writing the manuscript. IWS conceived and designed the research. JYN and JHY performed the data collection. IWS and JSK performed the statistical analysis and the interpretation of the data. IWS and SYY were involved in writing the manuscript. KHW and SYC had critically revised the manuscript. All authors read and approved the final manuscript.
